# The impact of ambient air pollution on suicide mortality: a case-crossover study in Guangzhou, China

**DOI:** 10.1186/s12940-016-0177-1

**Published:** 2016-08-30

**Authors:** Guo-Zhen Lin, Li Li, Yun-Feng Song, Ying-Xue Zhou, Shuang-Quan Shen, Chun-Quan Ou

**Affiliations:** 1 Guangzhou Center for Disease Control and Prevention, Guangzhou, 510440 China; 2 State Key Laboratory of Organ Failure Research, Department of Biostatistics, Guangdong Provincial Key Laboratory of Tropical Disease Research, School of Public Health, Southern Medical University, Guangzhou, 510515 China; 3 Intensive Care Unit, Guangdong No.2 Provincial People’s Hospital, Guangzhou, 510317 China

**Keywords:** Suicide, Mortality, Air pollution, Guangzhou

## Abstract

**Background:**

Preventing suicide is a global imperative. Although the effects of social and individual risk factors of suicide have been widely investigated, evidence of environmental effects of exposure to air pollution is scarce. We investigated the effects of ambient air pollution on suicide mortality in Guangzhou, China during 2003−2012.

**Methods:**

A conditional logistic regression analysis with a time-stratified case-crossover design was performed to assess the effects of daily exposure to three standard air pollutants, including particulate matter less than 10 μm in aerodynamic diameter (PM_10_), sulphur dioxide (SO_2_) and nitrogen dioxide (NO_2_), on suicide mortality, after adjusting for the confounding effects of daily mean temperature, relative humidity, atmospheric pressure and sunshine duration. Further analyses were stratified by season, gender, age group, educational attainment and suicide type.

**Results:**

Between 2003 and 2012, there were a total of 1 550 registered suicide deaths in Guangzhou. A significant increase in suicide risk were associated with interquartile-range increases in the concentration of air pollutant, with an odds ratio of 1.13 (95 % confidence interval (CI): 1.01, 1.27) and 1.15 (95 % CI: 1.03, 1.28) for PM_10_ and NO_2_ at lag 02, and 1.12 (95 % CI: 1.02, 1.23) for SO_2_ at lag 01, respectively. The suicide risks related to air pollution for males and people with high education level were higher than for females and those with low education level, respectively. Significant air pollution effects were found on violent suicide mortality and in cool season but not on non-violent suicide mortality or in warm season.

**Conclusions:**

Suicide risk was positively associated with ambient air pollution levels. This finding would provide important information for the health impact assessment of air pollution and for the development of effective strategies and interventions for the prevention of suicide.

## Background

Suicide was the second leading cause of death among those aged 15−29 years old globally and it was the 15th leading causes in the general population in 2012 [1]. There are more than 800 000 suicide deaths annually among which 75 % occur in low- and middle-income countries [1]. Because of the considerable premature deaths caused by suicide and the economic and psychological burden of suicide on family and the community, preventing suicide is a global imperative.

Suicide is multifactorially caused, such as individual’s physical and psychological factors, social, cultural and environmental factors. The risk factors identified may vary by geographical regions and populations [2, 3]. Previously, many individual risk factors have been identified and documented well, including some physical illnesses and mental disorders, economic difficulties, social isolation, disaster and physical abuse [1, 3]. There are increasing concerns about the effects of environmental risk factors on suicide. Suicide attempts and mortality show apparent seasonal variations [4–7], which are likely driven by environmental factors. Some time-series studies have shown the associations between suicide and various meteorological variables, such as mean temperature, diurnal temperature range and sunlight [8–11]. In previous epidemiological studies of health effects of air pollution on mortality, the analyses were usually restricted to non-external mortality on the assumption that there is no impact of air pollution on external mortality [12, 13]. However, it remains unknown whether this assumption is valid or not. In particular, suicide mortality comprises a considerable proportion of external mortality, while little is known about the potential impact of air pollution on suicide mortality. Recently, Bakian et al. [14, 15] provided new evidence of environmental impacts on suicide by examining the association with ambient air pollution in the US. Previously, two studies conducted in Korea and Taiwan have also reported acute effects of particulate matter [2, 8] on suicide mortality. Although these studies indicate that ambient air pollution may be an underexplored risk factor of suicide, the results varied among regions [16]. In addition, suicide shows seasonal fluctuations which may vary with gender and suicide methods (violent vs non-violent) [4, 7, 17, 18]. It is required to determine whether the effect of air pollution on violent suicide mortality is different from that on non-violent suicide mortality. More studies in other regions are required to confirm and further characterize the impacts of air pollution on suicide.

There is no study examining the air pollution-suicide association in mainland China. Guangzhou has experienced rapid economic growth and subsequent changes in culture and people’s emotional well-being during the last two decades, where suicide is an important public health problem. This study aimed to assess the effects of ambient particulate matter with an aerodynamic diameter of 10 μm or less (PM_10_), sulphur dioxide (SO_2_) and nitrogen dioxide (NO_2_) on suicide mortality and determine the potential effect modification by individual characteristics during the period of 2003–2012 in Guangzhou, China.

## Methods

### Data

During the study period, the coverage area of Guangzhou was gradually extended to the periphery from six original urban districts to ten districts and two nearby country-level cities. To keep the study area consistent, we only included six original central urban districts. According to China’s sixth population census, these six districts have an area of 1 166 km^2^ and have a population of 7.7 million permanent residents, accounting for 60.8 % of population in Guangzhou. Guangzhou Centre for Disease Control and Prevention provided all mortality registration records from 2003 to 2012 in Guangzhou. The causes of death were based on physicians’ death certificates and coded according to the International Classification of Diseases, tenth version (ICD-10). We extracted all individual suicide mortality data (ICD-10: X60–X84). In addition, we stratified data by age group (<65 and ≥65 years), gender, educational attainment (low: illiterate and primary education; high: secondary and higher education) and suicide methods (non-violent: X60–69; violent: X70–84).

Individual information for all deaths was matched to daily data of ambient air pollution and meteorological measures with the date of death. Daily concentrations of three criteria ambient air pollutants, including particulate matter less than 10 μm in aerodynamic diameter (PM_10_), sulphur dioxide (SO_2_) and nitrogen dioxide (NO_2_), were obtained from Guangzhou Bureau of Environmental Protection. The number of monitoring stations in Guangzhou increased gradually. In this study, we used the air pollution data of all seven fixed-site monitoring stations that have completed data during the study period of 2003–2012. These stations are distributed at five of six central urban districts under study [19, 20]. The average daily air pollutant concentrations of seven stations were computed. Daily data of meteorological measures were retrieved from China Meteorological Data Sharing Service System, including daily mean temperature, relative humidity, atmospheric pressure, and sunshine duration. Sunshine duration is defined as the total hours per day when direct solar irradiance exceeds a threshold value of 120 W/m^2^.

### Statistical analyses

We used a time-stratified case-crossover design to examine the association between air pollutant exposure and suicide mortality. All cases (i.e. suicide decedents) were served as his/her own control. Conditional logistic regression models were fitted to assess the effects of air pollution on suicide by comparing the levels of air pollutant concentrations between case periods and control periods. The day of suicide death was defined as case period, while control periods included all days that were from the same day of the week within the same month as the case period. The trend of suicide mortality was controlled for by the design since the case period and control period were very close. The potential confounding effects of holidays, daily mean temperature, relative humidity, atmospheric pressure and sunshine duration were controlled for by using an indicator variable for holidays and natural cubic spline functions of 6-day moving average for the current and previous 5 days for all meteorological factors. The corresponding degrees of freedom (*dfs*) were specified to be 6 for temperature and 3 for other meteorological factors, respectively [21]. To avoid collinearity, concentrations of three pollutants were included in regression models separately. We estimated separately the effects of air pollutants at single-day lag (from lag 0 to lag 7) and at multi-day lag (lag 01–lag 07). For example, lag 0 and lag 01 correspond to the concentration of pollutant on the suicide day and moving average for the suicide day and one previous day, respectively.

Further, we performed subgroup analyses by gender, age group, educational attainment, suicide type, and stratified analyses by season (cool season: October-March; warm season: April-September). The strength of association was measured as the odds ratio (OR) of suicide mortality associated with an interquartile-range (IQR) increase in pollutant concentrations.

All analyses were performed using R language version 3.2.1 (R Foundation for Statistical Computing, Vienna, Austria).

## Results

**Fig. 1:**
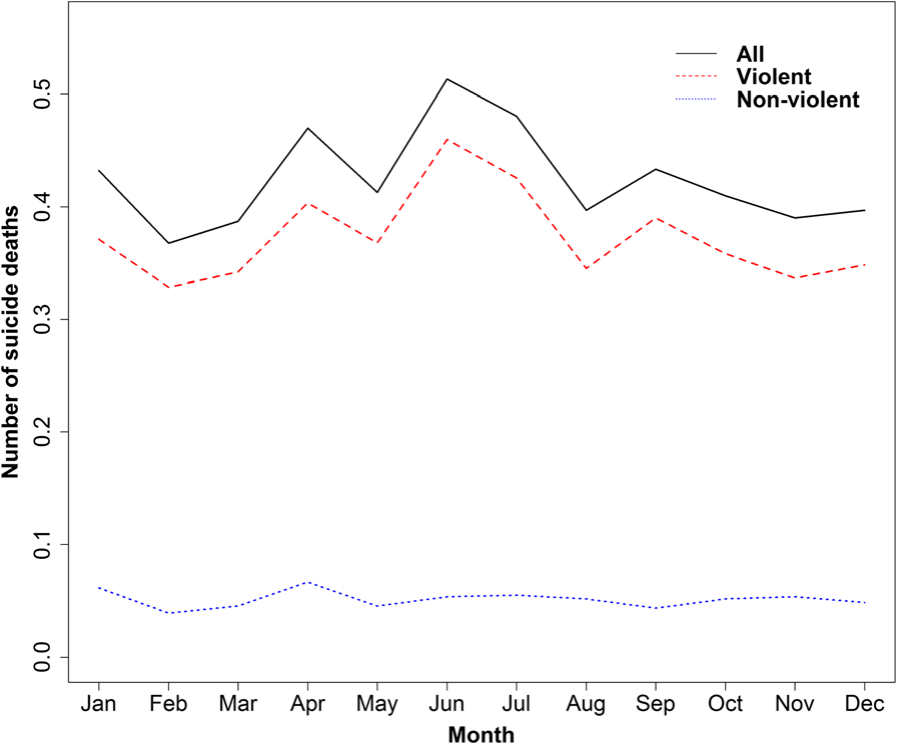
Mean monthly number of suicide deaths in Guangzhou, 2003-2012

During the study period from 2003 to 2012, there were a total of 1 550 registered suicide deaths with an average annual suicide rate of 3.3 per 100 000 population. Suicide accounted for 0.64 % of all registered deaths and 16.75 % of accidental deaths, respectively. 880 suicide deaths (56.8 %) were males with a 1.26:1 male-female ratio of suicide rate. 1 058 of suicide deaths (68.3 %) occurred among persons aged less than 65 years and 65.9 % were from those with high educational attainment. The majority of deaths (87.9 %) were due to violent suicide (Table 1). There were considerable seasonal variations of suicide with a peak in June and a trough in February. And violent suicide showed more apparent seasonality than non-violent suicide (Fig. 1).

**Table 1:** Demographic characteristics of suicide deaths in Guangzhou, China, 2003 to 2012

Factor	Groups	Suicide deaths (%)
Age group	<65 ages	1058 (68.3)
	≥65 ages	492 (31.7)
Gender	Male	880 (56.8)
	Female	670 (43.2)
Educational attainment^a^	Low	485 (34.1)
	High	939 (65.9)
Suicide type	Non-violent suicide	187 (12.1)
	Violent suicide	1363 (87.9)
Total		1550

^a^There were 126 subjects (8.1 %) whose educational attainments were missing, so we calculated the proportion as the number of each category divided by the sum of two education categories

**Fig. 2:**
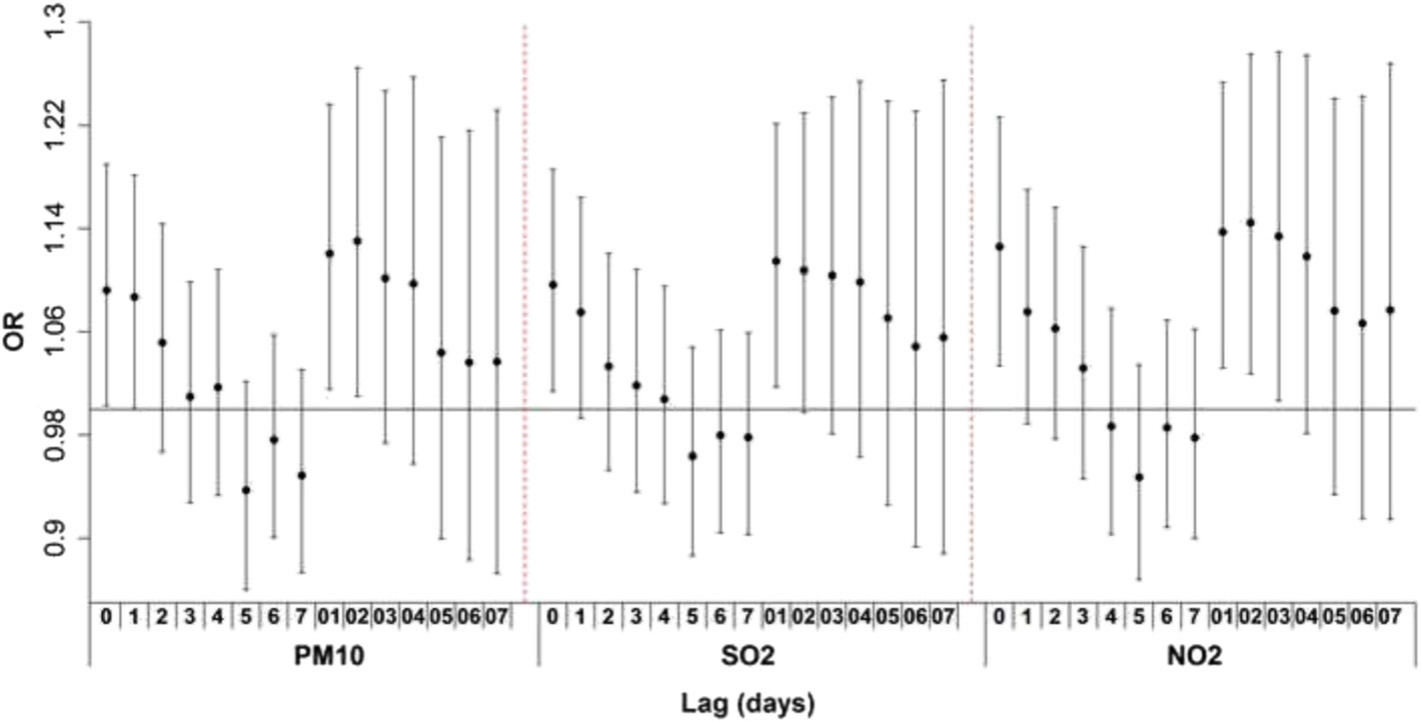
Lag structures of the odds ratio of suicide associated with interquartile-range increases in exposure to three air pollutants

Summary statistics of air pollution variables and meteorological measures used in this study were presented in Table 2. Guangzhou has a typical subtropical climate, with an average temperature of 22.6 °C and a range of 5.1 °C–34.2 °C during the study period. The mean concentrations of PM_10_, SO_2_ and NO_2_ were 79.6 μg/m^3^, 46.3 μg/m^3^ and 65.3 μg/m^3^, respectively. And they were consistently higher during the case period than the control periods. Daily concentration of PM_10_ was highly correlated with daily NO_2_ level with a Spearman correlation coefficient of 0.85 (*p* < 0.001). SO_2_ were also moderately correlated with PM_10_ (*r*_*s*_ = 0.64, *p* < 0.001) and NO_2_ (*r*_*s*_ = 0.65, *p* < 0.001). Figure 2 shows the effects of air pollution at a single lag day and the cumulative effects at multi-day lag. The effects gradually faded over the subsequent seven days. The largest effects were associated with the exposures to PM_10_ and NO_2_ at lag 02 before death and the exposure to SO_2_ at lag 01 before death. The following analyses reported the results for PM_10_ and NO_2_ at lag 02 and for SO_2_ at lag 01.

**Table 2:** Summary statistics for daily weather conditions and air pollutants in Guangzhou, China, 2003 to 2012

Variables	Frequency distribution					Mean	SD
	Min	25 %	Median	75 %	Max		
Mean temperature (°C)	5.1	18.2	24.2	27.7	34.2	22.6	6.3
Case periods	5.4	18.9	24.5	27.9	34.2	23.0	6.1
Control periods	5.1	18.5	24.7	27.9	34.2	22.9	6.3
Relative humidity (%)	20.0	65.0	74.0	82.0	99.0	72.4	13.0
Case periods	22.0	65.0	75.0	82.0	99.0	73.2	12.5
Control periods	23.0	66.0	75.0	82.0	99.0	72.8	13.0
Atmospheric pressure (hpa)	987.4	1002.5	1007.4	1013.0	1027.2	1007.7.	7.0
Case periods	987.4	1 001.9	1 006.8	1 012.7	1 025.2	1 007.2	7.1
Control periods	987.4	1 002.1	1 006.9	1 012.8	1 027.2	1 007.3	7.0
Sunshine duration (hours)	0.0	0.3	4.1	7.8	11.8	4.3	3.6
Case periods	0.0	0.3	3.9	7.6	11.8	4.2	3.6
Control periods	0.0	0.2	4.0	7.7	11.8	4.3	3.6
PM_10_ (μg/m^3^)	7.0	48.3	70.6	100.6	370.1	79.6	43.8
Case periods	7.0	48.6	71.7	103.6	370.1	81.2	44.2
Control periods	7.0	48.0	70.4	99.4	370.1	78.9	42.4
SO_2_ (μg/m^3^)	2.3	21.4	37.1	60.9	237.3	46.3	34.3
Case periods	3.0	23.1	38.5	64.6	226.1	48.3	35.3
Control periods	2.3	21.3	36.4	60.1	237.3	46.1	34.6
NO_2_ (μg/m^3^)	16.7	43.2	57.8	78.8	281.3	65.3	30.4
Case periods	16.7	43.7	59.4	79.9	254.7	66.6	31.3
Control periods	16.7	43.0	57.7	77.7	281.3	64.5	29.5

**Table 3:** The odds ratio of suicide mortality associated with interquartile-range increases in air pollutant levels and its 95 % confidence interval

Factors	PM_10_^a^	SO_2_^b^	NO_2_^a^
All	1.13 (1.01, 1.27)	1.12 (1.02, 1.23)	1.15 (1.03, 1.28)
Season			
Warm season	1.13 (0.91, 1.41)	1.05 (0.89, 1.23)	1.10 (0.88, 1.37)
Cool season	1.19 (1.04, 1.37)	1.18 (1.05, 1.33)	1.22 (1.07, 1.40)
Age group (years)			
< 65	1.10 (0.96, 1.25)	1.17 (1.05, 1.31)	1.17 (1.03, 1.33)
≥ 65	1.23 (0.99, 1.52)	0.98 (0.82, 1.17)	1.09 (0.88, 1.34)
Gender			
Male	1.20 (1.03, 1.39)	1.18 (1.04, 1.33)	1.23 (1.06, 1.42)
Female	1.06 (0.89, 1.26)	1.05 (0.91, 1.21)	1.05 (0.89, 1.24)
Educational attainment			
Low	1.13 (0.92, 1.39)	0.96 (0.82, 1.13)	1.08 (0.88, 1.31)
High	1.15 (1.00, 1.33)	1.22 (1.09, 1.38)	1.21 (1.05, 1.38)
Suicide type			
Non-violent	0.90 (0.65, 1.25)	0.80 (0.60, 1.06)	0.84 (0.61, 1.15)
Violent	1.17 (1.04, 1.32)	1.17 (1.06, 1.29)	1.21 (1.07, 1.36)

^a^The effect estimate at lag 02; ^b^ the effect estimate at lag 01

**Fig. 3:**
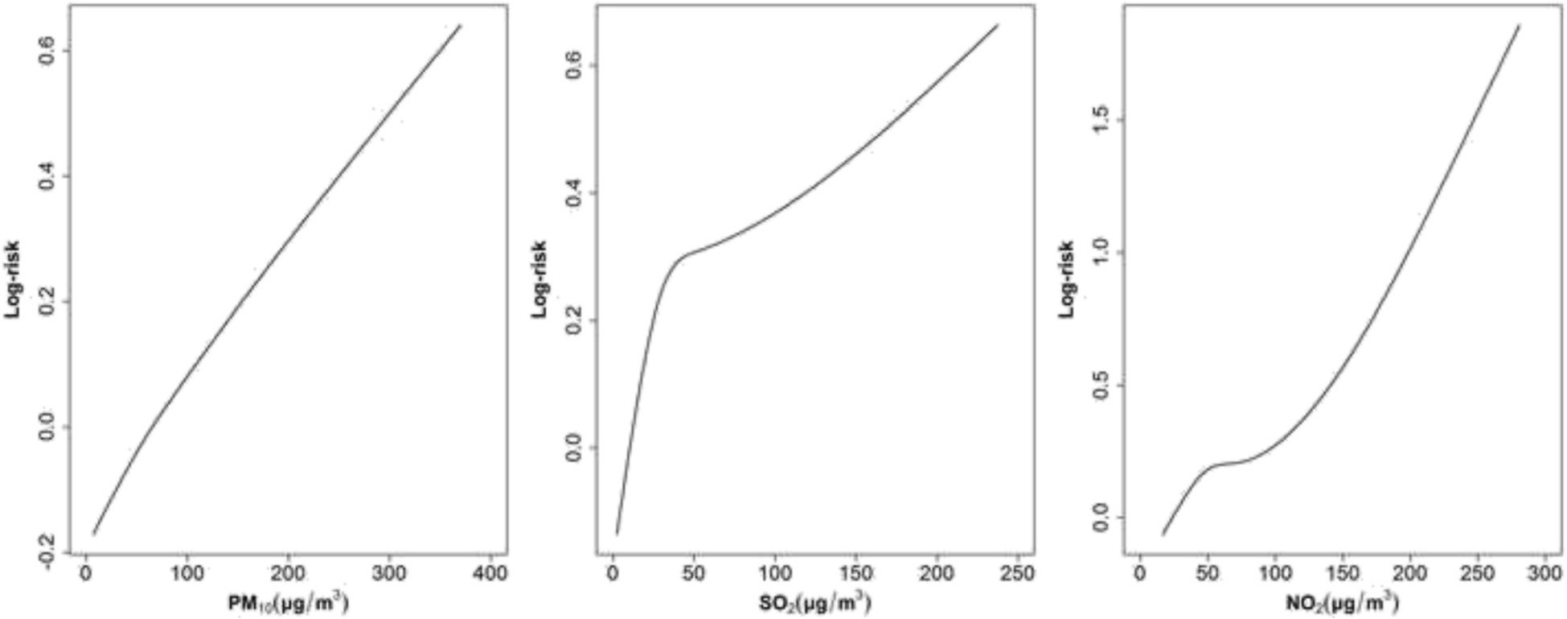
Dose-response relationships between daily number of suicide deaths and the concentration of PM_10_ and NO_2_ at lag 02, and SO_2_ at lag 01

The dose-response curves reveal that the risk of completed suicide generally increased with levels of air pollutant concentrations (Fig. 3). A significant increase in suicide mortality was associated with interquartile-range increases in air pollutant concentration, with an OR of 1.13 (95 % confidence interval (CI): 1.01, 1.27), 1.12 (95 % CI: 1.02, 1.23) and 1.15 (95 % CI: 1.03, 1.28) for PM_10_, SO_2_ and NO_2_, respectively. The effects of all pollutants were consistently statistically significant in cool season but not in warm season. Greater effects were observed in males and residents with high education level than in females and those with low education level, respectively. We observed statistically significant effects of SO_2_ and NO_2_ in persons less than 65 years but not in the elderly. There were statistically significant effects of all three air pollutants on violent suicide mortality but not on non-violent suicide (Table 3).

## Discussion

To the best of our knowledge, this is the first study in mainland China that reported significant detrimental effects of all three pollutants on completed suicide, with a 13 %, 15 % and 12 % increase in suicide risk associated with an IQR increase in PM_10_ and NO_2_ at lag 02, and SO_2_ at lag 01, respectively. A few literatures showed inconsistent results of air pollution effects on suicide. In Korea, Kim et al. [21] reported a maximum increase of 9.0 % and 10.1 % in suicide risk related to an IQR increase in PM_10_ at lag 02 and PM_2.5_ at lag 01. However, another meta-analysis in 16 Korean regions did not find evidence for the effects of gaseous pollutant (SO_2_, NO_2_ and carbon monoxide (CO)) on monthly suicide rate [16]. Recently, Bakian et al. [14] found an increased suicide risk of 20 % and 5 % associated with an IQR increase in NO_2_ at lag 03 and PM_2.5_ at lag 02 while no significant effects of PM_10_ and SO_2_ were found in Salt Lake Country, Utah. The effect estimates associated with an IQR increase in air pollutant concentrations reported in our study were, with the exception of the estimate for NO_2_ in Utah, greater than that reported in Utah and Korea, probably because of much higher levels and greater variations (i.e. greater IQR) of air pollution in Guangzhou.

Some specific mental and neurological disorders, such as depression and headache, have been linked to ambient air pollution [22, 23]. It is plausible that suicide as the most serious outcome of such disorders is adversely affected by air pollution. Mounting experimental evidence suggests that, in addition to cardiopulmonary system, the brain may be a target of air pollution. Inhaled concentrated ambient particulates (CAP), especially the ultrafine size fraction, may be able to translocate to the brain and produces neuroendocrine and neuropathological alterations, including elevated levels of oxidative stress and norepinephrinein in the brain, significant decreases in neurons from substantia nigral nucleus compacta and neuroinflammation [24–26]. Impaired short-term memory and behavior changes were observed in experimental animals exposed to CAP [27]. Experimental literatures also provide evidence for neurotoxic effects of SO_2_ exposure, such as oxidative stress and mitochondrial dysfunction in neuronal cells [28], neuronal insult and synaptic dysfunctions [29] and impairment of neuronal behavior in experimental animals [30]. Recently, an animal study reported that acute NO_2_ inhalation induced mitochondrial morphological changes in rat cortex and the abnormality of mitochondrial energy metabolism [31], which is involved in the pathogenesis of various neurological disorders.

Ambient air pollution can aggravate physical illness or discomfort and mental pain, subsequently inducing suicide. Consistent with previous studies on suicide mortality or all-cause mortality [13, 14, 21], we reported a latent period of 1–3 days between the time of exposure to air pollution and the time of peak effect. The lags of effect would earn time for early actions to prevent suicide. On heavily polluted days, we should increase awareness of preventing suicide and special attention should be paid to people at high risk such as those with prior mental disorders. It should be pointed out that the case period was defined as the date of suicide deaths because the information of the date of suicide attempt was unavailable in the mortality database. This may be a possible explanation for the lagged effect on suicide mortality since some suicide deaths did not happen on the current day of suicide attempt.

This study furthers our understanding of susceptible groups to the effects of air pollution. We observed greater air pollution-associated suicide risk among males and people under 65 years compared to females and people aged 65 or above. Similarly, another study based on a 1-year data from seven Korean cities also showed slightly stronger PM-suicide association in males and those aged 36–64 years [21]. It is possible that these subpopulations are more likely to work outdoors and experience greater exposure to ambient air pollution. Increasing concerns have been raised about the potential socioeconomic disparities in health effects of air pollution. Some studies reported higher mortality risk associated with air pollution in those with lower socio-economic conditions [13, 32]. The Korean study [21] reported the greatest air pollution effects on suicide among those of middle socioeconomic status. In this study, the education level was classified into two groups: high education and low education. The high:low education ratio of total suicide deaths was 2:1 and greater effect estimates (OR) of air pollution were found in those with high education level, suggesting that the majority of air pollution-associated suicide deaths were highly educated people. Pompili et al. [33] suggested that individuals with high educational attainment are possibly more prone to suicide risk when facing failures, public shame, and high premorbid functioning. Highly educated people commonly have higher expectation in some domains such as life quality, career advancement, etc.. For them any failures may cause adverse psychological reactions. Such psychological reaction may be heightened on polluted days. It is suggested that the presence of psychiatric illness increase the effects of air pollution on suicide [21]. Air pollution may aggravate depression symptoms and therefore increase suicide risk among high educated people. However, the prevalence of psychiatric disorders in suicide cases in different education groups was not investigated in this study. Further study with individual risks of suicide such as prior diagnosis of mental illness is helpful to clarify this hypothesis.

Kim et al. [21] and Bakian et al. [14] found stronger effects of air pollution in transition season (spring/fall) in two temperate regions. Literature shows the seasonality of air pollution effects may be different in subtropical regions. For example, Wong et al. [34] found greater effects of four pollutants (PM_10_, NO_2_, SO_2_ and ozone) on respiratory admissions occurred consistently in cool season in Hong Kong but in warm season in London, probably because of different seasonal pattern of air pollution levels between these two cities. Stronger effects of PM_2.5_ on mortality in cool season were also observed in another tropical city, Kaohsiung, Taiwan [35]. In this study in the subtropical city of Guangzhou, we found significant impacts of three pollutants on suicide mortality in cool seasons, while the impacts were not statistically significant in warm seasons. The air pollution in cool season is more severe than in warm season, so people inhale more air pollutants in cool season and the effect of air pollution on suicide mortality may be more apparent in this season. Second, it has been shown that influenza enhanced the effect of air pollution on health [36], whether the influenza aggravate the air pollution effects on suicide mortality in cool season in Guangzhou need to be further confirmed. Further, in the subtropical city of Guangzhou, the common use of air conditioner in summer makes people spend most of their time indoors, which partly prevent them from the exposure to air pollution. We found clear evidence for the effects of ambient air pollution on violent suicide but not for non-violent suicide, which may be due to the phenomenon of remarkable seasonal variations in violent but not non-violent suicide [17, 18]. The heterogeneity of environmental effects of ambient temperature on violent and nonviolent suicide was also observed previously, suggesting different causal pathways for these two suicide types [37]. De Vriese et al. [38] observed that the seasonal variations in long-chain poly-unsaturated fatty acids were significantly correlated with weekly number of violent suicide deaths but not with nonviolent suicide deaths.

Studying the association between air pollution and suicide provides a novel perspective for explaining the seasonal fluctuations and the regional disparities in suicide by the temporal and geographic variations of air pollution levels. We found ambient air pollution is an important environmental risk factor of suicide after adjusting for climatic factors. The finding suggests that a multisectoral public health approach, including the reduction of air pollution, is encouraged to develop or strengthen comprehensive suicide prevention strategies.

There are some limitations in this study. First of all, all data of suicide deaths were extracted from municipal mortality registry system and suicide cases were identified by death certificates. It is inevitable that some suicide deaths are missing or misclassified as some other causes of death in death certificates, leading to underreporting of the number of deaths. Our study using 10-year data did not reveal statistical significant effects of air pollution for some subgroups like females and the elderly; it is possible that the limited statistical power due to the small number of suicide deaths failed to detect the effects. In addition, we used the average level of air pollution measures collected at seven monitoring stations as a proxy of individual exposure, which would produce measurement errors in exposure and tend to bias the effects toward the null. We assessed the effects of three standard pollutants but did not consider other recognized air pollutants, such as ozone and PM_2.5_, because the data was not available during the study period. The three pollutants under study were significantly correlated. As in previous studies, we estimated the effects of each single pollutant separately. How to assess the overall effects of multiple pollutants after taking into account the collinearity is still an important issue to be addressed. Lastly, this study only examined the effects on completed suicide. Further investigation of air pollution impacts on suicide ideation and suicide attempts would help better understanding the role of air pollution in suicide. Meanwhile, it is notable that individual factors are undoubtedly main influencing factors of suicide. An investigation in Guangdong reported the main risk factors of suicide attempts included being female, anxiety, loneliness, and negative life events [39]. In this month-stratified case-crossover study, the individual risk factors are not changed in a month and therefore cannot confound the results of air pollution effects. However, it would be interesting to examine the interaction effect between environmental factors and traditional individual risk factors if data are available.

## Conclusions

We confirmed significant effects of ambient particulate pollutant and gaseous pollutants (SO_2_ and NO_2_) on suicide mortality in a subtropical city in China. Particularly, males and people with high education level were more susceptible to the effects. The findings would strengthen the evidence base for the deleterious effects of air pollution on suicide. Furthermore, the findings would broaden the scope of assessment of air pollution-mediated health hazards and provide important information for the development of effective strategies and interventions for the prevention of suicide.

## Abbreviations

CAP: Concentrated ambient particulates
CI: Confidence interval
ICD-10: International Classification of diseases, 10th version
IQR: Interquartile-range
NO_2_: Nitrogen dioxide
OR: Odds ratio
PM_10_: Particulate matter less than 10 μm in aerodynamic diameter
SO_2_: Sulphur dioxide
